# Blood-spinal cord barrier disruption in degenerative cervical myelopathy

**DOI:** 10.1186/s12987-023-00463-y

**Published:** 2023-09-25

**Authors:** Hyun Woo Kim, Hu Yong, Graham Ka Hon Shea

**Affiliations:** Department of Orthopaedics and Traumatology, LKS Faulty of Medicine, The University of Hong Kong, Hong Kong, China

**Keywords:** Degenerative cervical myelopathy, Blood-spinal cord barrier, Cervical decompression, Ischemia, Inflammation, Cell therapy, Gene therapy

## Abstract

Degenerative cervical myelopathy (DCM) is the most prevalent cause of spinal cord dysfunction in the aging population. Significant neurological deficits may result from a delayed diagnosis as well as inadequate neurological recovery following surgical decompression. Here, we review the pathophysiology of DCM with an emphasis on how blood-spinal cord barrier (BSCB) disruption is a critical yet neglected pathological feature affecting prognosis. In patients suffering from DCM, compromise of the BSCB is evidenced by elevated cerebrospinal fluid (CSF) to serum protein ratios and abnormal contrast-enhancement upon magnetic resonance imaging (MRI). In animal model correlates, there is histological evidence of increased extravasation of tissue dyes and serum contents, and pathological changes to the neurovascular unit. BSCB dysfunction is the likely culprit for ischemia–reperfusion injury following surgical decompression, which can result in devastating neurological sequelae. As there are currently no therapeutic approaches specifically targeting BSCB reconstitution, we conclude the review by discussing potential interventions harnessed for this purpose.

## Introduction

Degenerative cervical myelopathy (DCM) is the commonest cause of spinal cord dysfunction in developed countries due to age-related changes within the cervical spinal canal [1, 2]. Chronic mechanical compression of the spinal cord results from encroachment by surrounding structures. Neurological manifestations include sensory impairment, decline in hand dexterity, limb weakness, gait instability, and even tetraplegia if left untreated [3, 4]. Average age at diagnosis is estimated to be 65 and the disease classically exhibits a steady, stepwise deterioration with stable intervening periods [5–8]. There remains inadequate awareness of DCM amongst the public as well as primary health care providers, resulting in delay in diagnosis or misdiagnosis [1, 9]. Although operative treatment via cervical decompression is effective, full recovery is uncommon whilst non-operative treatment modalities show limited clinical efficacy [10–12]. As a disease with a substantial and increasing global burden, understanding the mechanisms contributing to disease pathology is essential to advancing diagnosis, treatment, and recovery. In this review, we delve into the significance of blood-spinal cord barrier (BSCB) disruption to DCM pathophysiology, whilst proposing novel BSCB-based treatment strategies that may be beneficial to management.

## The blood-spinal cord barrier in health and disease

### BSCB architecture

The BSCB is generally considered to be an extension of the blood brain barrier (BBB). They share the same ultrastructural arrangement, although differences in morphology and function do exist as will be discussed in the following section. The BSCB is comprised of capillary endothelial cells and its accompanying basement membrane, pericytes, and astrocytic end-feet. Endothelia over the BSCB are distinct in being non-fenestrated. They form tight junctions (TJs) with adjacent cells [13–16], and compared to the endothelia of other tissues possess a low density of pinocytic vacuoles and a high number of cytosolic mitochondria [17, 18]. The basement membrane is composed of laminins, collagen IV isoforms, nidogens, and heparan sulphate proteoglycans (HSPGs) to form a three-dimensional matrix [19, 20] and is maintained by endothelial cells as well as embedded pericytes [21, 22]. Astrocytes encircle endothelia, contributing to the basement membrane, and enwrap neuronal synapses to enable neurovascular coupling [19, 20, 23]. This arrangement allows for neurohumoral regulation of blood flow, controlling the inflow and efflux of nutrients, energy stores, metabolites, and toxins within the neurovascular unit (NVU) [24–27]. The spinal cord perivascular space is continuous with the subarachnoid space [16]. At the capillary level, the basal lamina of endothelial cells is in direct contact with the glia limitans. Upon inflammation, basal lamina may separate into two layers, forming a transient perivascular space or “loop” that facilitates leukocyte infiltration [15, 20].

**Fig. 1:**
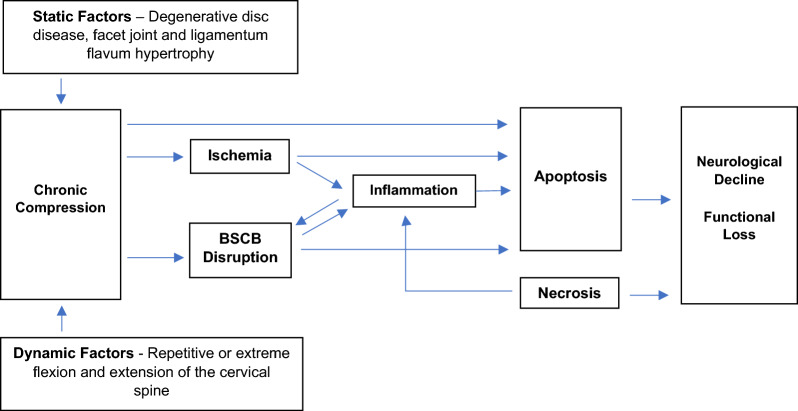
Pathophysiology of degenerative cervical myelopathy

### BSCB vs BBB

Animal studies indicate that the BSCB has increased permeability to serum biomolecules, cytokines, and growth factors, such as mannitol, inulin, interferon (IFN) α/γ, and nerve growth factor (NGF) compared to the BBB [28–30]. This may be explained by lower expression of tight and adherent junction proteins in spinal cord endothelial cells compared to brain endothelial cells, which is associated with increased paracellular transport [31–33]. Mouse pericyte number and coverage within the BSCB is reduced in comparison to the BBB, which is associated with increased endothelial transcytosis and barrier permeability [34, 35]. Genes associated with astrogliosis i.e. GFAP, IL-6, and STAT3, are expressed at higher levels in mouse spinal cord astrocytes compared to astrocytes in the brain. Enhanced GFAP expression may help the spinal cord to withstand bending and torque [36], allowing the BSCB to tolerate mechanical stresses associated with vertebral movement. Significantly higher mitochondria content was observed in rat spinal cord endothelial cells compared to endothelial cells of the cerebral cortex and cerebellum, which may indicate more robust active transport systems and diminished vesicular transport [17, 37].

### BSCB disruption in traumatic spinal cord injury

In acute spinal cord injury (SCI), primary injury causes immediate physical disruption of the BSCB [38, 39]. Edema of spinal cord parenchyma is an early macroscopic manifestation of barrier disruption that is correlated with contusion force [40–43]. Hemolysates from extravasated erythrocytes and serum macromolecules such as serine proteases perpetuate neuroinflammation as well as oxidative stress [43, 44]. Even after filtration of high molecular weight proteins (> 10 kDa), serum contents cause apoptosis of cultured spinal cord neurons, likely due to the presence of neurotoxic depolarizing agents such as glutamate [41, 42]. Animal models have revealed that cord edema peaks at Day 5 post-injury [38], whilst permeability to large serum macromolecules resolves around 2 weeks post-SCI. Nevertheless, permeability to smaller molecules persists especially in areas with microglial aggregates, implying lasting alterations to BSCB permeability [45, 46]. There is evidence indicating that BSCB alteration is a key factor in the pathogenesis of post-SCI syringomyelia, which is characterized by the formation of cystic cavities over the lesion epicentre [46–48].

### BSCB disruption in neurodegenerative and autoimmune diseases

BSCB disruption is also a key factor contributing to the pathogenesis of amyotrophic lateral sclerosis (ALS), a progressive and fatal neurodegenerative disorder affecting upper and lower motor neurons located in the brain and spinal cord [49, 50]. In necropsy specimens of spinal cords obtained from ALS patients, there was evidence of cytotoxic lipofuscin deposits within the capillaries, decreased endothelial expression of TJs, and increased infiltration by leucocytes, erythrocytes, and serum macromolecules [51–54]. Pericyte and astrocyte end-feet degeneration were also observed at sites corresponding to vessel rupture [49, 55, 56]. Likewise, in the SOD1 mouse ALS model, impaired BSCB function and vascular pathology preceded motor neuron degeneration, which was evidenced by vascular leakage and loss of TJs throughout the spinal cord [52, 57]. Histological and imaging studies on experimental autoimmune encephalomyelitis (EAE) animal models of multiple sclerosis (MS) showed evidence of BSCB disruption during early disease, sometimes preceding neurological manifestations [58–61]. However, clinical studies on the impact of BSCB disruption in MS pathogenesis is lacking. BSCB disruption in DCM, traumatic SCI, and neurodegenerative disease (using ALS as an example) is compared in Table 1.

**Table 1:** Comparison of BSCB Pathology in Traumatic SCI, neurodegenerative disease, and DCM

		Traumatic SCI	Neurodegenerative disease (ALS)	Degenerative cervical myelopathy
Pathophysiology of BSCB breakdown and reconstitution	Etiology	• Acute blunt / penetrating trauma	• Chronic inflammation	• Chronic mechanical compression
	Onset / Progression of BSCB Damage	• Rapid onset and progression	• Insidious onset and progression	*Ongoing compression*: • Gradual and dependent on clinical severity *Reperfusion injury after decompression:* • Rapid onset, subacute injury
	BSCB reconstitution	• Early; complete recovery by 3–4 weeks	• Unresolved, unless treatment of underlying disease	*Ongoing compression*: • Unresolved due to persistent, progressive compression *After decompression*: • Reconstitution in DCM of mild to moderate clinical severity, prolonged reconstitution / persistent deficits with severe clinical severity
Histological and molecular features	Presence of edema	• Present at acute stage, resolves by 1–2 weeks	• Not present	•Infrequent, observed only in severe DCM
	Cystic cavitation	• Present in 1–5 % of SCI patients [62]	• Not present	• Infrequent, observed only in severe DCM [63]
	MMP9 expression	• Present	• Present	• Present
	Erythrocyte or tracer dye extravasation	• Extensive extravasation upon spinal cord hemorrhage	• Continuous low-level extravasation, leading to parenchymal accumulation of cytotoxic iron and lipofuscin [64, 65]	• Erythrocyte extravasation unknown • Evans Blue extravasation observed during ongoing compression in experimental animal models
	Others	• Extravasation of hemolysates and proteases • Prolonged BSCB permeability at sites with microglial clusters	• Enlarged perivascular space with infiltrating leukocytes • Decreased TJs • BSCB impairment marks early-stage ALS and precedes clinical presentation	• Swollen endothelial cells filled with caveolae-like vesicles • Abnormal TJs with large gaps, thickened basement membrane • Thickened basement membrane

## Pathophysiology of degenerative cervical myelopathy

### Animal models of DCM

In addition to necropsy specimens, animal model correlates of DCM have been invaluable to understanding disease pathophysiology, and in highlighting differences from acute traumatic SCI [66]. Acute traumatic SCI models result in immediate neurological deterioration (i.e. via cord contusion, distraction, and transection) [67], whilst animal models mimicking DCM cause progressive neurological compromise following chronic compression. Murine models are most frequently utilized. *Twy/twy* mice possess an autosomal recessive, non-sense mutation at the *Npps* (nucleotide pyrophosphatase) gene locus [68]. Failure of the *Npps* enzyme to produce inorganic phosphatase, a major inhibitor of calcification, causes progressive soft-tissue calcification and bone mineralization to compromise the cervical canal [69]. Limitations to this model exist, in that the site of maximal compression is within the upper cervical spine which is atypical for DCM, and decompression of the ossified spinal canal is technically infeasible. In rats, implantation of expandable polymers dorsal to the spinal cord following a laminectomy is a common experimental setup, and decompression may be modelled via polymer removal. Implant positioning, size, and swell rate are important variables to control [70]. The rate and region of cervical compression is better controlled in larger animal models following polymer implantation, and there are means to mimic ventral compression for example via screw insertion from the anterior vertebral body, although larger animals are costly, may not be readily available, and present more ethical concerns [70–76].

### Mechanical compression

DCM is a multifactorial disorder instigated by static and dynamic mechanical compression of the cervical spinal cord. Causes for static compression include degenerative disc disease (DDD) and congenital cervical stenosis. DDD also results in cervical microinstability, and over time this causes secondary spondylotic changes such as cervical facet and posterior longitudinal ligament hypertrophy to further mechanical compression [77]. Dynamic factors, for example in occupations requiring prolonged and exaggerated movement of the cervical spine, may further mechanical insult [78, 79]. Biological processes resulting from chronic compression include tissue ischemia, BSCB disruption, and neuroinflammation, culminating in the loss of neurons and glia. These have been summarized in Fig. 1 and will be discussed in further detail below.

**Fig. 2:**
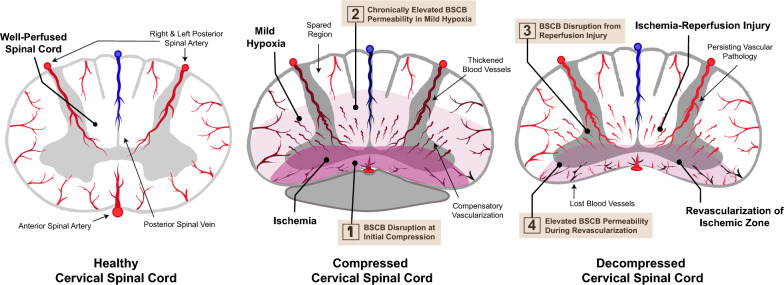
Blood spinal cord barrier disruption during and after cervical decompression in degenerative cervical myelopathy. Spinal cord perfusion is maintained by the anterior spinal artery, left/right posterior spinal arteries, and their associated veins. Chronic cervical cord compression (schematically represented as anterior compression alone) causes pathological changes to these supplying vessels and disrupts the BSCB. Spinal cord tissues remain hypoperfused despite vascular remodeling. Following surgical decompression, there is sudden restoration in blood flow but the BSCB remains hyperpermeable. This predisposes the cord to reperfusion injury and impairs neurological recovery, although the underlying mechanical compression has been relieved. Bright red and blue colors denote healthy blood vessels, while dark red and blue colors indicate blood vessels with compromised blood supply. Pink-shaded regions indicate mildly hypoxic regions, while darker pink-shaded regions indicate ischemic regions with severe ischemia

### Ischemia

Spinal cord perfusion is compromised in DCM as evidenced by distorted anterior spinal and radicular arteries upon necropsy specimens [78]. Cord perfusion may be further compromised by vessel wall thickening and hyalinization [80–82]. Evidence of ischemic injury is manifest early in medial grey and white matter tracts, which are supplied by terminal branches of the anterior spinal artery [80]. Corresponding to this topography, the lateral corticospinal tract is first to be affected in DCM [83–86]. In the polymer-implanted rat DCM model, a significant reduction in the number of blood vessels in white and grey matter of the cervical spinal cord was observed [75]. Ischemic injury precipitates apoptosis and inflammation, which can proceed to necroptosis as cord compression worsens [66, 87].

### BSCB disruption

It remains uncertain whether mechanical compression is an independent factor for BSCB disruption, or whether its effects are exerted secondary to compression-induced ischemia. Regarding pathophysiology of the latter, spinal cord ischemia causes pericytes located at the BSCB to express hypoxia inducible factor-1 (HIF-1). HIF-1 dilates blood vessels and disrupts endothelial TJs, thereby increasing BSCB permeability [88]. Mild chronic spinal cord hypoxia in mice was sufficient to induce vascular leakage, resulting in extravasation of neurotoxic serum macromolecules and activation of microglia [89]. Another mechanism for BSCB breakdown is the upregulation of matrix metallopeptidase 9 (MMP-9) within the spinal cord of DCM patients and animal model correlates [90–92]. MMP9 is a proteolytic enzyme which is expressed in neutrophils and endothelial cells, and by degrading the basement membrane, compromises BSCB integrity [91]. BSCB disruption causes an influx of inflammatory cells [87], exposes the spinal cord to neurotoxic serum contents, impairs clearance of metabolic waste, and result in the accumulation of protein aggregates which generate oxidative stress [93, 94]. This instigates further injury to the BSCB, forming a vicious cycle.

### Inflammation

Inflammation in DCM occurs consequent to chronic ischemia and BSCB disruption [77]. In animal models, CNS hypoxia induces neuroglia to release proinflammatory cytokines such as IL-1β, IL-6, IL-8, FasL, and TNF-α [90, 95–97]. These cytokines, together with nuclear factor kappa B (NF-κB), MMP-2, and urokinase-type plasminogen activator (u-PA) are also detected in DCM necropsy specimens [92, 98]. Proinflammatory cytokines increase macrophage recruitment, infiltration, and activation, as evidenced by a 12-fold increase in Iba1 expression in DCM spinal cords compared to controls [75]. In the experimental hyperostotic (*twy/twy*) DCM mouse model, macrophages within the cord parenchyma are predominantly of the cytotoxic M1 phenotype [99].

### Apoptosis

There are multiple disease processes leading to cellular apoptosis in DCM. Firstly, proinflammatory cytokines and neurotoxins can directly bind to cell death receptors upon neurons and glia [41, 100–104]. Secondly, loss of cellular homeostasis from ischemia can activate apoptotic pathways in neural cells by causing membrane depolarization, Ca^2+^ influx, and glutamate release [105]. Lastly, mechanical compression can cause cytoskeletal degradation and result in calponin-mediated neuronal apoptosis [106]. Evidence for activation of TNF-α, MAPK, and FasL mediated apoptotic pathways have been demonstrated in both animal models and human specimens. In the *twy/twy* mouse, TNF-α signaling mediated oligodendrocyte apoptosis [107], as did mitogen-activated protein kinase (MAPK) pathways involving ASK1, JNK, and p38 [108]. In a necropsy study, FasL-mediated apoptosis was implicated in mediating neuronal and oligodendrocyte apoptosis, and FasL neutralization led to increased cell survival and improvement in functional recovery in the animal model correlate [90].

## BSCB dysfunction during different phases of DCM

### Ongoing mechanical compression

Literature indicates that the extent of BSCB dysfunction is correlated with DCM disease severity. CSF/serum ratios of albumin and IgG in mild-to-moderate DCM patients are only marginally increased compared to normal patients [109]. A systematic review on the disease progression of DCM patients concluded that 38–80% of patients with mild DCM and managed non-operatively improved neurologically or remained unchanged, suggesting that an equilibrium or reconstitution of BSCB function can occur within this patient subset [110]. In severe DCM, CSF/serum ratios of albumin and IgG are over two-fold higher when compared to normal controls, indicating significant barrier compromise [109]. An imaging correlate of BSCB breakdown is T1-weighted hyperintensity with Gd-DTPA enhancement, which indicates spinal cord edema [111–115]. In the rat insertable polymer DCM model, BSCB histopathology was characterised by swollen endothelial cells filled with caveolae-like vesicles, abnormal TJs with large gaps, pericyte enlargement, swollen perivascular astrocytes with disrupted mitochondria, and thickened basement membrane [74, 116, 117]. Rat disease models also exhibit increased extravasation of serum Evans Blue, markedly impaired angiogenesis, and decreased endothelial barrier protein (EBA) immunopositivity [74, 75].

### BSCB function following surgical decompression

Patients with severe or rapidly progressive DCM are prone to exhibit poor neurological recovery [118–120]. A particular disastrous manifestation following surgery is known as White Cord Syndrome (WCS). WCS is so-named due to the de novo appearance of white T2 hyperintense lesions upon post-operative MRI images that accompany neurological deterioration with onset typically within 24-h of decompression [121, 122]. It is hypothesized that WCS occurs due to reperfusion injury to the spinal cord, which initiates inflammatory and apoptotic cascades [123, 124]. Whilst WCS only affects 0.3% of patients, the recovery trajectory of many more is likely affected by a leaky BSCB.

### Is the BSCB reconstituted following surgical decompression?

Clinical evidence suggests that BSCB function is largely intact in mild DCM [109]. Additionally, a cohort with an averaged JOA score of moderate severity demonstrated barrier reconstitution at 3-months post-decompression, with JOA improvement correlating with the extent of barrier recovery [125]. In severe DCM, chronic BSCB disruption is suggested by T1-weighted hyperintensity with Gd-DTPA enhancement persisting for months, even after the onset of neurological recovery [112, 126, 127]. In the insertable polymer rat model, hypervascularization of spinal cord regions adjacent to the compression region was observed with a concomitant increase in Evans Blue dye extravasation [74]. Therefore, neovascularization and altered blood flow in response to chronic ischemia may attenuate capacity for vascular remodelling following decompression [74, 80, 82, 128, 129]. Figure 2 is a schematic diagram postulating the status of the blood spinal cord barrier and spinal cord vasculature during compression and decompression, when the BSCB fails to be reconstituted.

**Fig. 3:**
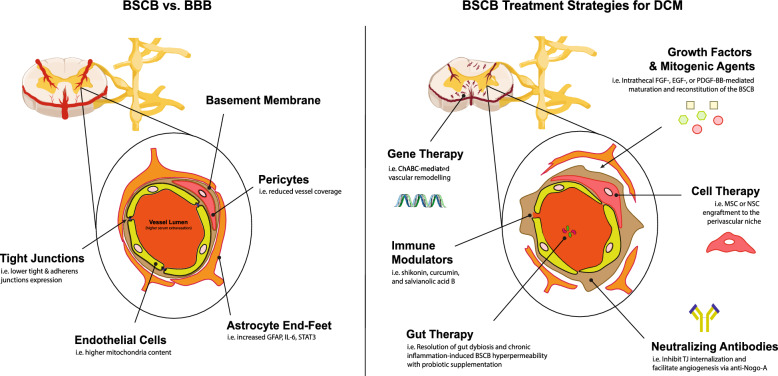
Components of the blood spinal cord barrier (BSCB) and therapeutic strategies for BSCB reconstitution in degenerative cervical myelopathy (DCM). Left panel—At the BSCB, the presence of non-fenestrated endothelial cells establishes tight junctions that heavily restrict paracellular transport. At the capillary level, the basement membrane is closely associated with astrocyte end-feet, resulting in the elimination of the perivascular space. Pericytes, embedded within the basement membrane, assume a crucial role in facilitating endothelial cell maturation, supporting the basement membrane, and potentially modulating blood flow. Disruption of the BSCB integrity is characterized by the thickening or swelling of the basement membrane, endothelial cells, pericytes, and astrocytes. Deterioration of the tight junctions leads to the leakage of serum contents into the surrounding tissues. Inflammation leads to the transient formation of the perivascular space at the capillary level, thereby enabling leukocytes infiltration into the spinal cord parenchyma. Right panel—The treatment modalities mentioned in this figure are elaborated in the main text

## Potential therapeutic strategies to ameliorate BSCB Disruption

### An overview of DCM management at present

At present, patients with radiological cervical canal stenosis and moderate to severe neurological impairment, often defined as having a JOA score of 13/17 or less, are recommended to receive surgery. Improvement in neurological function has been reported after surgery for over 70% of patients, most notably over the upper limb, followed by the lower limb and sphincters [130]. Nevertheless, patients with severe DCM often report residual sensory deficits or limb spasticity and incoordination [131]. Factors most strongly predictive of neurological outcomes following surgery include preoperative neurological severity and duration of symptoms [132]. Late-onset neurological deterioration may also occur despite adequate mechanical decompression [133, 134]. There is insufficient evidence to support the long-term efficacy of non-operative management approaches for DCM such as physiotherapy, nutritional supplementation, use of analgesics and non-steroidal anti-inflammatory drugs (NSAIDs), cervical steroid injections (CSIs), traction, and acupuncture [10, 135–137]. Few DCM-related therapeutics have undergone clinical testing. Of note, a Phase III trial on the safety and efficacy of peri-operative riluzole has been recently completed for DCM patients undergoing decompression [138]. Riluzole is a neuroprotective agent approved for clinical use in ALS, which acts by mitigating glutamate-induced excitotoxicity in the CNS [139, 140]. Although riluzole did not improve neurological outcomes, reduction in neck pain was observed. There remains a clinical necessity to identity adjuncts to surgical decompression especially amongst patient groups with i) mild disease yet to require surgery, ii) non-recovery / deterioration after surgery, and iii) at-risk groups for poor surgical outcomes. As summarised in Table 2 and Fig. 3, we subsequently discuss potential strategies to promote BSCB reconstitution in DCM.

**Table 2:** Summary of potential BSCB treatment strategies for degenerative cervical myelopathy

Category	Treatment	Mechanism	Evidence of BBB/BSCB support
Cell therapy	Mesenchymal stem cell (MSC) transplantation	Paracrine trophic support, immunomodulation, engraftment into BSCB, and angiogenesis [142]	AD-MSCs engraft into blood vessels to promote angiogenesis and recruit pericytes in SCI rats [149] UC-MSCs and BM-MSCs engraft into vascular wall, preserve BBB, and support angiogenesis in ischemic stroke (IS) mice [235–238] BM-MSCs reduce EB extravasation and increase filament density in astrocytes in LPS-treated rats [239] AD-MSCs alleviate brain edema in intracerebral hemorrhage (ICH) mice [240]
	Neural stem cell (NSC) transplantation	Same as above [141, 143]	F-NSCs associate with cerebral vasculature, decrease MMP9 expression, decrease IgG and biotin extravasation, and preserve TJs in IS mice [141, 241] aSVZ-NSCs robustly become VLA-1 + pericytes and modulate CNS inflammation and leukocyte trafficking in MS mice [151] hESC-NSCs and F-NSCs predominately become pericytes in a mouse model of PD [152]
	Cell-derived exosomes	Release of trophic factors and miRNA that preserve and stimulate growth of cells of the BSCB, modulate inflammation, and facilitate angiogenesis, leading to BSCB support [242–245]	Mouse Mouse pericyte-derived exosomes reduce lesion size, ameliorate microcirculation, reduce EB extravasation, preserve TJs, and reduce edema in SCI mice [242] Human BM-MSCs-derived exosomes enhance neurorestoration in a porcine model of TBI by reducing brain swelling, decreasing intracranial pressure, and supporting BBB integrity evidenced by decreased albumin extravasation and increased TJ expression [246] Rat BM-MSCs-derived exosomes increase TJ expression, promote remyelination, and decrease MMP-9 in IS mice [156]
Gene therapy	AAV-delivery of growth factors	Expression of growth factors by BSCB cells, angiogenesis, and neuronal sprouting	AAV-delivery of FGF, EGF, and GDNF revascularizes glial scar and forms neurite growth-supportive environment [177]
	ChABC	Vascular and astrocytic remodelling	ChABC attenuates hypertrophy of blood vessels and basement membrane pathology while facilitating vascular remodelling [178]
	siRNA	Inhibition of proinflammatory cascades	siRNA-mediated inhibition of P2X purinergic receptor and Tim-3 attenuate neuroinflammation and edema in ICH mice and reduce neuropathic pain in rats [183–186]
Growth factors & mitogenic agents	Fibroblast growth factors (bFGF)	Reduction of autophagy and ER stress, maturation of BSCB, and proliferation of endogenous NSCs [159]	bFGF improves functional recovery in SCI rats via attenuation of autophagy and BSCB reconstitution evidenced by decrease in EB and dextran extravasation, inhibition of MMP9, and preservation of TJs [161–164] bFGF enhances proliferation and maturation of NSCs and protects the BBB by upregulating TJs in traumatic brain injury (TBI) rats [165, 166]
	Platelet derived-growth factor BB (PDGF-BB)	Increase pericyte coverage, reduce pathological activation of pericytes, and maturation of the BSCB [157, 158]	Intrathecal delivery of PDGF-BB in PD mice increases pericyte coverage in the dorsolateral striatum and reduces pathological activation of pericytes, leading to behavioral recovery [167] Intraspinal delivery of PDGF-BB in SCI mice increases TJ expression, reduces autophagy, increases revascularization, and reduces EB extravasation, leading to improvement in recovery [168]
	Epidermal growth factor (EGF)	Proliferation and migration of NSCs, protection of BSCB from oxidative stress, maturation of the BSCB	EGF reduces EB extravasation and preserves TJs via PI3K pathway, induces migration of ependymal cells and astrocytes to the injured spinal cord, and decreases oxidative stress and apoptosis, leading to improved functional recovery in SCI rats [169–171]
	Adropin	Proliferation and maturation of endothelial cells [160]	Adropin decreased endothelial cell monolayer permeability to dextran and decreased macrophage infiltration in vitro via NO release [172] In ICH mice, intraperitoneal adropin led to decreased hematoma, brain edema, and EB and IgG extravasation, leading to improvement in behavioral outcomes via Notch1 signaling [173, 247]
Molecular inhibitors & antibodies	Anti-Nogo-A	Neutralization of Nogo-A, a membrane protein expressed by oligodendrocytes and neurons that inhibit neurite outgrowth and angiogenesis [187–189]	Expression of TJ and BBB permeability is restored in anti-Nogo-A treated IS mice, especially upon co-administration of VEGF, leading to improved revascularization of the peri-infarct cortex [192]
	Imatinib	Inhibition of PDGF-CC signaling [248]	Intraperitoneal injection of imatinib preserves BBB integrity in ICH mice, evidenced by decreased brain edema, EB extravasation, and MMP-9 activity [249] In MS mice, oral imatinib led to decreased dextran extravasation and immune cell trafficking [250] Oral imatinib inhibits oxidative stress response and modulates neuroinflammation, leading to BSCB support in SCI rats, shown by preservation of TJs, reduced IgG and albumin extravasation, and prevention of pericyte detachment from the blood vessel wall [193, 194]
	4-Phenylbutyric acid (PBA)	Attenuation of ER stress by acting as chemical chaperone [195]	PBA treatment leads to reduced EB extravasation, increased pericyte coverage, and preservation of TJ and microvessels in SCI rats via modulating expression of ER stress markers [195, 196, 251]
	Salubrinal	Deactivation of ER stress pathway via inhibition of PP1α phosphatase [252]	Salubrinal treatment for a mouse model of TBI increases PDGF-BB expression in neurons while decreasing microglia activation and EB extravasation [197] In SCI rats, Salubrinal protects neurons and oligodendrocytes from apoptosis, leading to functional recovery [198]
Immune modulators	Infliximab	Inhibition of TNF-α	Infliximab significantly ameliorates endothelial necroptosis and BBB disruption, as shown by MRI-imaging and decreased EB extravasation in IS mice [202]
	Calpastatin	Inhibition of calpain (calcium-dependent proteolytic system) [253]	Calpastatin decreases caspase-mediated cell apoptosis in SAH mice, leading to reduced EB extravasation and decreased brain edema volume [204]
	Shikonin	Attenuation of NK-κB signaling and ER stress	Shikonin reduces spinal cord edema and prevents apoptosis in SCI rats, leading to increased motor recovery [205]
	Curcumin	Attenuation of TNF-α signaling [254]	Curcumin reduces EB extravasation and increases TJ expression via attenuation of TNF-α signaling, promoting motor recovery in SCI rats [207]
	Salvianolic Acid B	Attenuation of TNF-α signaling and oxidative stress [255]	Salvianolic acid leads to decrease in spinal cord edema, EB extravasation, and preservation of TJs, due to attenuation of oxidative stress and inflammation in SCI rats and rat spinal cord ischemia–reperfusion induced neuronal injury [208, 256]
Alternative therapies	Vitamin B12, B6, Folate	Metabolism of neurotoxic homocysteine associated with BBB breakdown [217, 257]	Enhanced amyloid clearance and pericyte rescue in Alzheimer's disease (AD) mouse model [257] Improved serum/CSF ratio of albumin and cognitive function in hyperhomocysteinemia patients with mild cognitive impairment [217]
	Omega-3 Fatty Acids	Reduction of inflammation-induced MMP-9 and -6 activity and BBB hyperpermeability [219]	Decreased extravasation of serum tracers and preservation of TJs in ischemic brain injury, AD, and post-operative delirium mouse models [258–260] High serum omega-3 levels correlate with improved BBB determined by contrast-enhanced MRI [220]
	Gut therapy	Prevention of systemic infiltration of neurotoxin bacteria and metabolites [225]. Promotion of SCFAs production [226]	Probiotic and micro-derived methylamines led to restored TJs and BBB integrity to EB in aged and stressed mice [227, 228, 230]
	Photobiomodulation	Maturation of BSCB cells and pericyte mobilization [232–234]	Decreased edema and serum extravasation in IS mice [234] Preservation of retinal capillaries and decreased extravasation in diabetic mice [261] Increased cerebral perfusion in Alzheimer’s Disease patients [262]

*AD* Alzheimer’s Disease, *AD-MSCs* adipose-derived mesenchymal stem cells, *aSVZ-NSCs* adult subventricular zone-derived neural stem cells, *ALS* amyotrophic lateral sclerosis, *BBB* blood–brain barrier, *BSCB* blood spinal cord barrier, *BM-MSCs* bone marrow-derived mesenchymal stem cells, *CSF* cerebrospinal fluid, *EB* Evans Blue, *F-NSCs* fetal-derived neural stem cells, *ICH* intracerebral hemorrhage, *IS* ischemic stroke, *LPS* lipopolysaccharides, *MMP* matrix metallopeptidase, *MS* multiple sclerosis, *NO* nitric oxide, *NSCs* neural stem cells, *PD* Parkinson’s Disease, *PTSD* post-traumatic stress disorder, *SAH* subarachnoid hemorrhage, *SCI* spinal cord injury, *SCAFs* short chain fatty acids, *TBI* traumatic brain injury, *TJs* Tight Junctions, *UC-MSC* umbilical cord-derived mesenchymal stem cells, *VEGF* vascular endothelial growth factor

### Cell therapy

Delivery of cells such as mesenchymal stem cells (MSCs) or neural stem cells (NSCs) into the spinal cord parenchyma may facilitate BSCB recovery via direct engraftment, trophic support, and immunomodulation [141–143]. NSCs and MSCs express PDGF which is a key marker delineating capillary pericytes [144–148]. Transplanted NSCs have been demonstrated to engraft into the perivascular niche and differentiate into PDGFRβ + CNS pericytes, and in doing facilitate angiogenesis, suppress neuroinflammation, and induce NVU maturation [141, 149–152]. Pericytes are descended from the neuroectoderm during development, as are a subpopulation of MSCs [153], thereby explaining their emergence from transplanted progenitors [144, 154, 155]. Exosomes provide an alternative to whole cells in facilitating BSCB reconstitution. As an example, bone marrow MSC-derived exosomes significantly increased TJ expression, promoted remyelination, and decreased production of MMP-9 [156] in a diabetic stroke model. Future cell-based studies focusing on BSCB pathology and reconstitution are essential to establish preclinical efficacy.

### Growth factors and mitogenic agents

Growth factors and mitogenic agents can support the BSCB by inducing proliferation and maturation of cells composing the BSCB, such as pericytes, astrocytes, and endothelium, thereby facilitating reconstitution [157–160]. Such restorative factors include platelet derived-growth factor-BB (PDGF-BB), fibroblast growth factors (FGF), epidermal growth factor (EGFs), and adropin [161–173]. Despite promising results from preclinical studies and early clinical trials, translation of bFGF therapy into spinal cord diseases has been slow [159]. Translational research into EGF and PDGF-BB for spinal cord disorders is nascent, with a few preclinical studies conducted demonstrating its efficacy [168, 169]. Since growth and mitogenic factors have diverse biological effects, further in vivo studies are required to characterize their effect on the BSCB.

### Gene therapy

Studies utilizing gene therapy-based approaches to treat diseases affecting the spinal cord have aimed to replenish neuroglial populations and replace absent or dysfunctional genes [174–176]. These have provided indirect evidence for the potential of gene therapy to facilitate restoration of the BSCB. In the context of acute SCI, AAV delivery of combined growth factors (FGF, EGF, and GDNF) to the lesion core resulted in an increase in basement membrane-associated laminin expression [177]. Chondroitinase ABC (ChABC) is an enzyme known for its ability to induce axonal sprouting in SCI by degrading chondroitin sulphate proteoglycans (CSPGs). ChABC has also been shown to promote vascular remodelling and attenuate secondary injury from neuroinflammation thereby presenting a candidate for gene therapy [178]. Several other siRNA-based therapies aimed at reducing neuroinflammation may also prevent or ameliorate BSCB injury [179–182]. Key mediators of proinflammatory cascades such as P2X-purinogenic receptors or toll-like receptor 4 (TLR-4) are ideal targets for siRNA-mediated silencing that could protect the BSCB and improve neurological recovery [180, 183–186].

### Molecular inhibitors and neutralizing antibodies

Inhibitors to BSCB-destabilizing factors and ER stress-induced apoptosis are amongst promising therapeutic agents that have been shown to reduce BBB / BSCB damage. One such agent is anti-Nogo-A neutralizing antibody. Nogo-A is a myelin-associated inhibitor that may be neutralized with an antibody to improve neurite outgrowth following spinal cord injury [187–189]. Interestingly, Nogo-A signalling via S1PR2 receptor activation and downstream RhoA/ROCK activation compromised vascular integrity by causing internalization of TJs and loosening of the endothelial lining [190–192]. Thus, anti-Nogo antibodies may also be effective in protecting the BSCB. Another drug candidate is imatinib, a receptor tyrosine kinase (RTK) inhibitor commonly used in cancer treatment and known to suppress PDGF-CC signalling, which plays a key role in BSCB disruption mediated by inflammation and oxidative stress [193, 194]. Other therapeutic candidates acting via inhibition of endoplasmic reticulum (ER) stress include 4-phenylbutyric acid (PBA) and salubrinal, which preserved endothelial cell survival and TJ integrity in mice subject to traumatic SCI [195–198].

### Immune modulators

Inflammation is a pathophysiological feature of DCM that results in cytotoxicity and compromises BSCB function. There are many clinical studies regarding the perioperative use of dexamethasone to reduce neurological complications in cervical spine surgery [199–201] but not BSCB disruption per se. Inhibiting proinflammatory cascades via attenuation of TNFα signaling and calpain with infliximab and calpastatin respectively restored BBB permeability and promoted endothelial cell survival and TJ expression in mouse models of subarachnoid hemorrhage (SAH) and traumatic brain injury (TBI) [202–204]. Compounds derived from natural herbs that possess anti-inflammatory or antioxidant activity, such as shikonin, curcumin, and salvianolic acid B have also demonstrated efficacy in rat models of SCI [205–208]. Arachidonic acid pathway attenuation [209–211], monoacylglycerol lipase (MAGL) inhibition [212, 213], and inhibition of complement C5a [214–216] are also potential pharmacological targets as alternatives to more established anti-inflammatory agents such as corticosteroids, riluzole, and NSAIDs.

### Other approaches

Studies on the BBB have demonstrated that nutritional remedies such as vitamins B and D [217, 218], omega-3 fatty acids [219, 220], and antioxidants such as glutathione and polyphenols [221–223] have a positive effect on barrier health. Gut microbiota is increasing being recognized as a key regulator of BBB function [224]. Dysfunction of gut microbiota can lead to the disruption of the gut-vascular barrier (GVB), leading to infiltration of bacteria and toxic metabolites into the bloodstream that induces chronic inflammation and NVU hyperpermeability [225, 226]. Lack of diversity in gut microbiome can also negatively impact BBB health, potentially due to reduced short-chain fatty acids (SCFAs) and production of beneficial microbes, which protect the NVU from oxidative stress [226]. Although these studies have only focused on the BBB, the same therapeutic principles apply to the BSCB, especially in patients with a dysregulated gut-brain axis [227–230].

Photobiomodulation (PBM), also referred to as transcranial low-level laser therapy (LLLT), is an experimental light therapy that has undergone clinical trials for stroke, TBI, and neurodegenerative disorders such as Alzheimer’s disease and Parkinson’s disease [231]. The mechanism of PBM is attributed to be via cytochrome C oxidase, a photoreceptor in the mitochondria that upon activation can promote proliferation and maturation of cells composing the BSCB [232]. Recently, PBM has been shown to increase pericyte mobilization and to support the BBB in stroke models [233, 234].

## Conclusion

BSCB disruption is increasingly recognized as a cause for neurological decline in disease affecting the spinal cord. Our review highlights the preclinical and clinical evidence for BSCB breakdown in DCM and identifies therapeutic strategies that may facilitate neurological recovery by means of BSCB reconstitution. Future studies should be performed upon representative animal models to characterise BSCB breakdown in moderate to severe DCM, and thereafter, to evaluate the efficacy of the aforementioned treatment modalities. Protection and regeneration of the dysfunctional BSCB in DCM provides a promising direction for future study as neurological and functional deficits often remain despite best available treatment.

## Data Availability

Not applicable.
